# A live-imaging protocol for tracking intestinal stem cell divisions in the *Drosophila melanogaster* pupal midgut

**DOI:** 10.1016/j.xpro.2023.102749

**Published:** 2023-12-01

**Authors:** Song Wu, Ruizhi Tang, Benjamin Ohlstein, Zheng Guo

**Affiliations:** 1 Department of Pharmacology, Bioengineering and Food College, Hubei University of Technology, Wuhan 430068, China; 2 Department of Medical Genetics, School of Basic Medicine, Institute for Brain Research, Tongji Medical College, Huazhong University of Science and Technology, Wuhan 430022, China; 3 Children’s Research Institute and Department of Pediatrics, University of Texas Southwestern Medical Center, Dallas, TX, USA; 4 Cell Architecture Research Center, Huazhong University of Science and Technology, Wuhan 430030, China

**Keywords:** Cell Biology, Cell culture, Developmental biology

## Abstract

Establishing a long-term *ex vivo* observation of the intestinal stem cell (ISC) is crucial to help understand the formation and homeostasis of the intestinal epithelium. Here, we present a protocol for tracking the division of *Drosophila* pupal ISCs during pupal midgut development. We describe steps for dissecting, mounting, and live imaging the pupal midgut. We then detail procedures for fluorescence quantification of each cell. This protocol can be applied to other fluorescently tagged proteins.

For complete details on the use and execution of this protocol, please refer to Wu et al.^1^

## Highlights

• Protocol for tracking the division of *Drosophila* pupal intestinal cells
• Dissection of the pupa to obtain the pupal midgut
• Mounting and live imaging of the pupal midgut and protein quantification

## Abstract (editor highlights)

Publisher’s note: Undertaking any experimental protocol requires adherence to local institutional guidelines for laboratory safety and ethics.

## Graphical abstract

**Figure:**
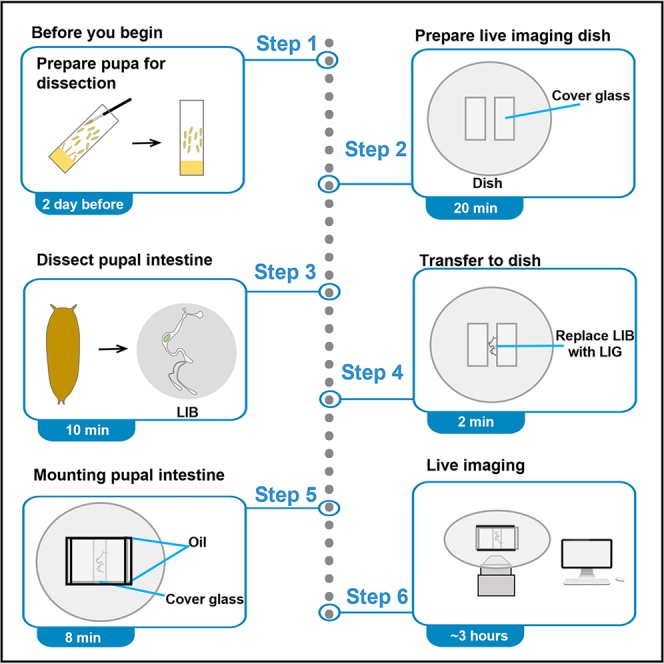

## Before you begin

The protocol below uses *Drosophila* pupae with the following genotype: *esg-Gal4, 10xUAS-Myr::tdTomato/+; Pros::GFP/+*. Red *10xUAS-Myr::tdTomato* is expressed in *esg*^+^ progenitor cells^2^ and green *Pros::GFP* is expressed in pupal enteroendocrine (EE) cells.^1^^,^^3^ *esg-Gal4,tub-Gal80*^*ts*^*, 10xUAS-Myr::tdTomato/+; Pros::GFP/+,* enables *10xUAS-Myr::tdTomato* and other UAS driven construct expression under the control of temperature.^4^ To study real-time expression and localization of other proteins in the midgut, fluorescently tagged proteins can be constructed using the knock-in method.^5^ In the study by *Wu* et al., *UAS-RNAi* lines were crossed into this system to knock down genes of interest exclusively in *esg*^+^ progenitor cells. To inactivate *Gal80*^*ts*^ and activate UAS-mediated gene expression, experimental samples were kept at 29°C for 2 days prior to experimentation. Note that pupal development is approximately 1.3 times faster at 29°C than at 25°C.^6^

### Preparation of specific stage pupae for experiments

**Timing: 2 days before**

**Figure 1:**
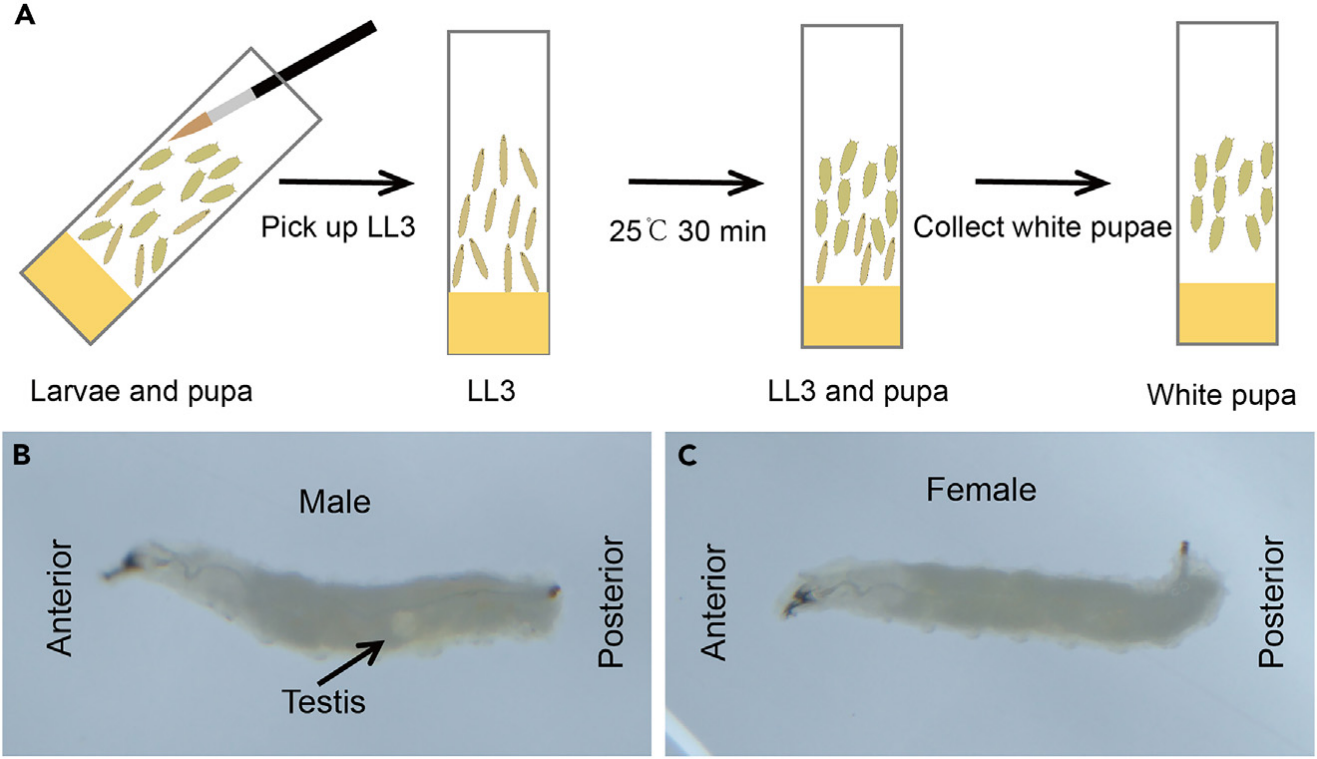
Collect white pupae (A) Collect LL3 and transfer to new vials. Store these LL3 vials at 25°C and collect white pupae (0 h APF) from LL3 vials every half hour. Transfer these pupae to new vials and make note of when pupae were collected (Figure 1A). (B) A testis (white transparent dot structure indicated by the arrow) on the mid-posterior part of a male LL3. (C) A female LL3.

***Note:*** Staging pupae is necessary to obtain reproducible results. In this protocol, we perform experiments using pupae between 46 h and 52 h after puparium formation (APF) at 25°C (Figure 1A).

1. Pick up late third instar larvae (LL3, stage during which the larva wanders out of the food and climbs) with a brush, and transfer them to new vials of standard cornmeal food, with about 30 larvae in each vial and kept at 25°C.
2. Collect white pupae (0 h APF) from LL3 vials and place these pupae in a vial every half hour, and note when these pupae were collected. Keep the vials containing pupae at 25°C (Figure 1A).

Culture flies at 25°C on standard cornmeal food,^1^ and transfer to new vials every 2 days to control the number of eggs. Female LL3 are selected as follows: LL3 are transferred to PBS with a brush. Under the microscope, white transparent dots (testis, arrowhead in Figure 1B) are present on both sides of the posterior part of male larvae, but not in female larvae (Figure 1C).

## Key resources table

**Table:** 

REAGENT or RESOURCE	SOURCE	IDENTIFIER	
**Chemicals, peptides, and recombinant proteins**			

4% Formaldehyde	Sigma-Aldrich	F8775	
DAPI	Sigma-Aldrich	D9542	
Schneider’s *Drosophila* medium	Gibco, Thermo Fisher Scientific	21720024	
FBS	Gibco, Thermo Fisher Scientific	10091148	
Insulin	Sigma-Aldrich	I0305000	
Gelatin	Sigma-Aldrich	G2500	
NaOH	Sinopharm Chemical Reagent	10019318	
Halocarbon oil 27	Sigma-Aldrich	H8773	
Type DF immersion oil	Zeiss	1111807	

**Experimental models: Organisms/strains**			

*yw*, *hs-Flp*; *esg-Gal4*, *10XUAS-Myr::tdTomato*; *pros::GFP/TM6B.Tb*	This paper	N/A	
*yw*, *hs-Flp*; *esg-Gal4*, *Tub-Gal80*^*ts*^, *10XUAS-Myr::tdTomato*; *pros::GFP/TM6B.Tb*	This paper	N/A	

**Software and algorithms**			

ImageJ	NIH	http://adm.irbbarcelona.org/image-j-fiji	
Photoshop CC	Adobe	http://www.adobe.com	
Photoshop Illustrator CS6	Adobe	http://www.adobe.com	
Prism 6	GraphPad	https://www.graphpad.com/	
OriginPro	OriginLabs	https://www.originlab.com/	

**Other (Except the lumox dish 50, all the other materials can be substituted with appropriate materials)**			

Cover glass	Citotest	80340-1130	
Lumox dish 50	Sarstedt (Critical)	15090935	
Forceps	Dumont	#5 or #55	
Metal bath	Scilogex	HB-120s	
Glass cutter	Airuize	6050	
Brush	Fandi	000200	
Dissecting plate	Xingrui	CZ3457	
Zeiss LSM 800 confocal microscope	Zeiss Microsystems	N/A	
63x/1.4 NA oil immersion objective	Zeiss Microsystems	N/A	

## Materials and equipment

### Stock solutions

#### Preparation of live imaging Buffer (LIB) used for pupal midgut dissection

**Timing: ∼20 min**

This step is performed in a sterile laminar flow hood at 25°C. Add 8.5 mL Schneider’s *Drosophila* medium and 1.5 mL FBS to a 15 mL sterilized centrifuge tube, and then add 2 mg human insulin and mix well, adjusting the pH to 7.0 with 1 M NaOH. Since 500 μL of LIB is an appropriate volume for a live imaging experiment, transfer 5 mL of LIB to ten 1.5 mL centrifuge tubes containing 500 μL each. The remaining 5 mL LIB is used to prepare the Live Imaging Gel (LIG).

***Note:*** The ten 1.5 mL centrifuge tubes containing 500 μL each can be stored at 4°C and used for one week.

#### Preparation of Live Imaging Gel (LIG) used for pupal midgut cultivation

**Timing: ∼10 min**

Add 0.5 g gelatin to the 5 mL LIB and heat on a metal bath at 50°C for 5 min. After the gelatin has melted, transfer the LIG to ten tubes of 500 μL each and store at 4°C.

***Note:*** This medium is critical for maintaining stem cell survival in the *Drosophila* pupal gut during time-lapse imaging. Aliquots of this medium should be stored at 4°C and can be used for one week.

## Step-by-step method details

### Preparation of live imaging dish

**Timing: ∼20 min**

**Figure 2:**
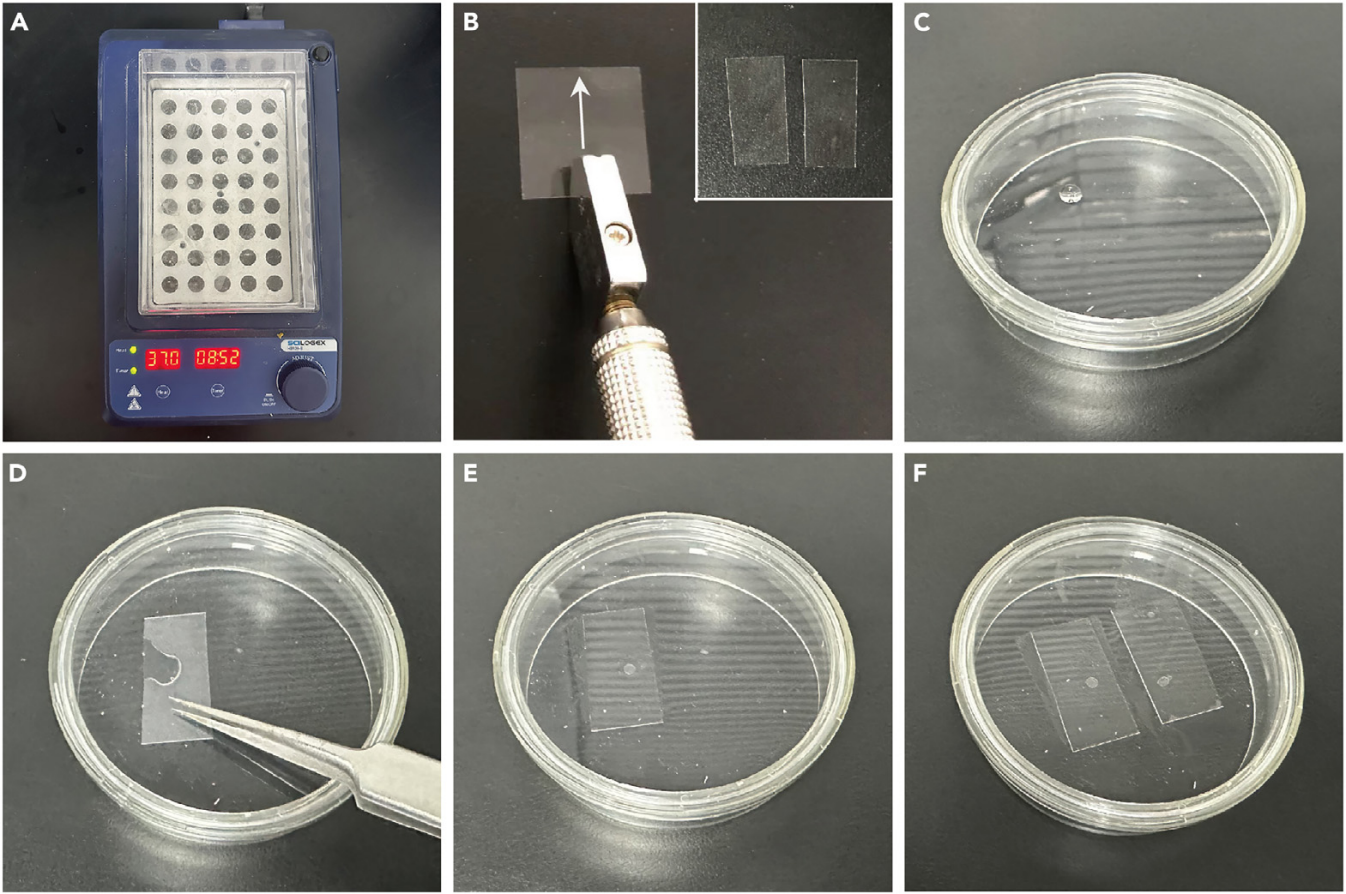
Prepare live imaging dish (A) Melt the LIG by heating it on the metal bath at 37°C for 10 min. (B) Cut a 22 mm × 22 mm coverslip into two pieces from the middle. (C) Drop 10 μL of melted LIG onto the dish. (D and E) Quickly cover the LIG with a pre-cut coverslip. (F) Repeat steps (C-E) and cover the other pre-cut coverslip, spacing the two coverslips approximately 5 mm apart.
2. Using a glass cutter, cut a 22 mm × 22 mm coverslip into two pieces from the center (Figure 2B).

***Note:*** The thickness of our coverslips is about 0.14 mm, and the coverslips are cut to make room for the dissected intestine. The coverslip can be cut with a glass cutter, or simply break the coverslip in half by hand (be careful not to injure your hand, and some of the coverslip may break).

1. Turn on the metal bath and heat to 37°C to melt the LIG by heating it on the metal bath for 10 min (Figure 2A). The temperature used to melt the LIG may increase to 40°C to prevent the LIG from solidifying too quickly.

3. Pipette 10 μL of melted LIG into the lumox dish 50 and quickly cover it with an 11 mm × 22 mm coverslip (Figures 2C–2E).
4. Repeat step 3 to place another coverslip, keeping the two coverslips approximately 5 mm apart (Figure 2F).

***Note:*** Providing an ideal environment for the intestine is critical for prolonging the survival time of the dissected pupal guts, and the live imaging dish we prepared here is air-permeable and nutritious, which is beneficial for the development of the dissected guts.

### Transfer the stage pupa to a dissecting microscope

**Timing: ∼5 min**

**Figure 3:**
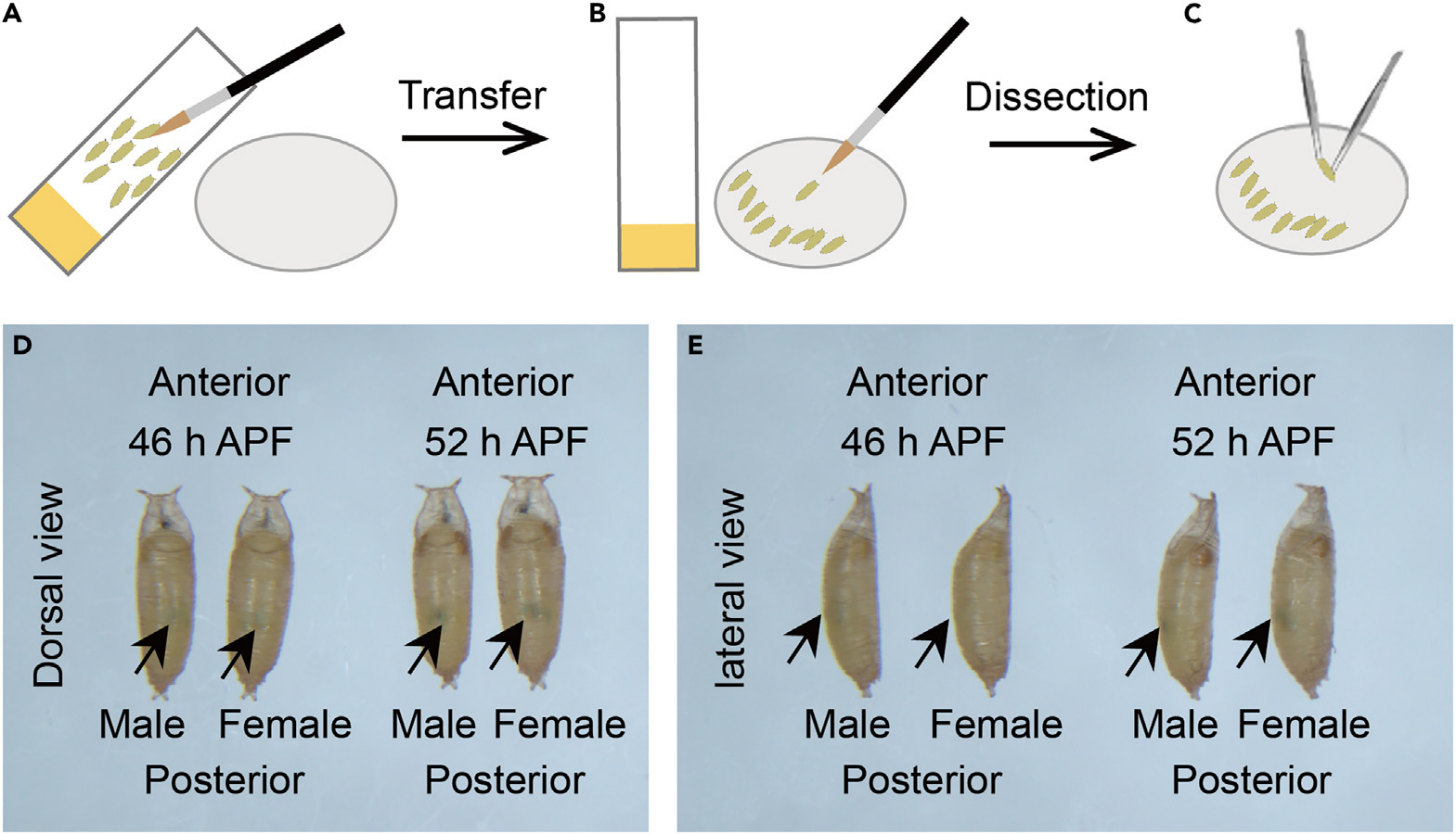
Prepare pupae for dissection (A) Gently lift the staged pupae from the side of the vial. (B) Transfer these pupae to the dissecting plate. (C) Pupae prepared for dissection under a dissecting microscope. (D and E) Dorsal and lateral views of male and female pupae at 46 h APF and 52 h APF (arrow indicates meconium). Typically, males have a darker yellow tail color and are smaller individuals compared to female pupae of the same developmental stage.
6. Transfer these pupae to the dissecting plate (Figure 3B).
7. Place the pupae under a dissecting microscope (Figure 3C).

***Note:*** We can get the exact developmental time of the pupae we want to study by timing from the white pupae. Since the developmental stages of the pupae are different at different times, we can also determine if the pupae are developing normally by the morphology (e.g. 46 h APF *Drosophila* eyes are yellowish, while at 52 h APF eyes deepen in color in the *yw, hs-Flp; esg-Gal4, 10XUAS-Myr::tdTomato; pros::GFP/TM6B*.*Tb*) (Figures 3D and 3E).

5. Use the brush to gently pry several properly staged pupae from the side of the vial (Figure 3A).

### Dissect the pupa to obtain the pupal midgut

**Timing: ∼5 min**

**Figure 4:**
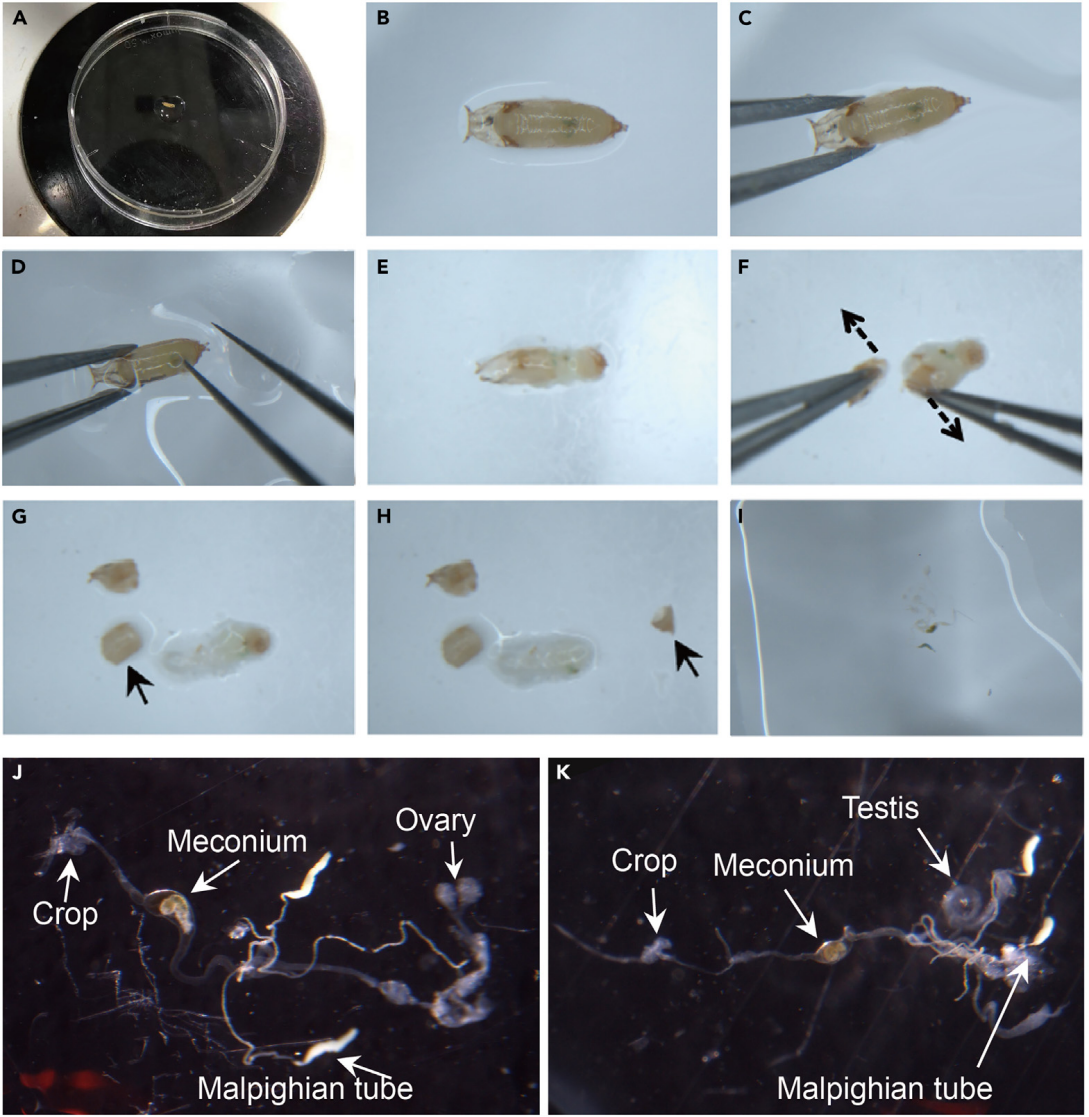
Dissect pupal midgut (A) Add 40 μL of LIB to the center of a dissection plate and transfer the pupa into the LIB. (B) Zoom view of a pupa ready for dissection. (C) Using a dull flat-ended forceps to gently grasp the anterior part of the pupa. (D) Using another pair of fine-tipped forceps, with the forceps open, press down very gently on the top of the meconium. (E) From the insertion position, divide the pupa into two parts, taking care not to pull the midgut apart. (F) Remove the head of the pupa by holding the head and neck of the pupa with two pairs of forceps and pulling in opposite directions (as indicated by the arrow). (G) Peel off the anterior shell. (H) Peel off the posterior shell. (I) Gently rinse the midgut by aspirating LIB through a 200 μL pipette to flush the midgut out of the pupal stage tissue. (J) A dissected female midgut. (K) A dissected male midgut.
9. Under the dissecting microscope, with the anterior of the pupa facing left if dissecting right-handed (right if dissecting left-handed), zoom in to enlarge the view of the pupa (Figure 4B).
10. Gently grasp the pupa from the anterior part using one pair of blunt, flat-ended forceps (Figure 4C).
11. Using another pair of fine-tipped forceps, with the forceps open, gently insert one tip of the forceps onto the top of the meconium (Figures 3D, 4D, and 4E).
12. Once the tip is inserted approximately one-third to one-half of the way down the pupa, pull back to expose the midgut, being careful not to break it.
13. Remove the head of the pupa by holding the head and neck of the pupa with two pairs of forceps respectively and pulling in opposite directions (arrow indicated in Figure 4F).
14. Use the flat-ended forceps to gently press down on the surface of the pupa to hold it in place, and use the fine-tipped forceps to peel off the anterior shell (Figure 4G).
15. Repeat step 14 to peel off the posterior shell (Figure 4H).
16. Gently rinse the midgut by aspirating LIB through a 200 μL pipette to flush the midgut out of the pupal stage tissue. At this point the midgut is attached to the crop and reproductive system (Figure 4I).

***Note:*** When using a new strippette tip, the midgut can easily adhere to the tip of the strippette. Washing the strippette tip 3 times with LIB before starting the experiment can greatly reduce the occurrence of this situation.

***Note:*** This method is specific for dissecting pupae between 46 h and 52 h APF. For dissecting other developmental stages: See troubleshooting 1.

8. Add 40 μL of LIB to the center of a dissecting plate and transfer one pupa into the LIB under a dissecting microscope (Figure 4A).

### Mount the pupal midgut on the live imaging dish

**Timing: ∼10 min**

**Figure 5:**
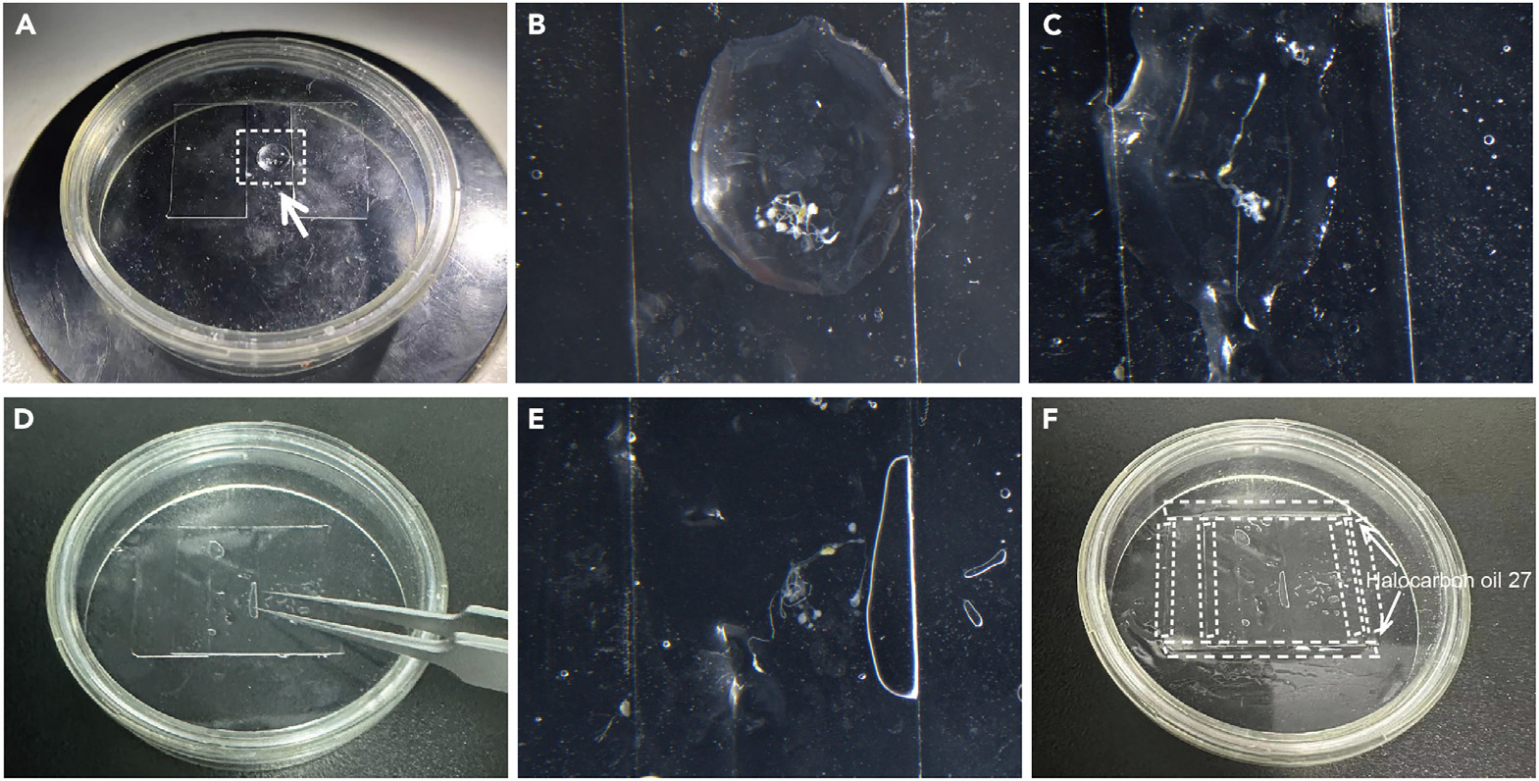
Mount of pupal midgut (A) Transfer of the dissected midgut to the live imaging dish. (B) Zoom view of the area indicated by the arrow in (A). (C) Replace LIB with LIG. (D) Add LIG between the two 11 × 22 mm coverslips and cover the midgut with the 22 × 22 mm coverslip and press it with forceps to stabilize it. (E) Zoom view of the midgut mounted in (D), an air bubble under the slide but not on the midgut does not affect the survival and imaging of the midgut. (F) Seal these coverslips with halocarbon oil 27 (dashed lines indicate the range of oil).
19. Add 10 μL 37°C LIG to the transferred midgut, gently mix with LIB, aspirate and discard 10 μL of the mixed liquid. Adjust the position of the midgut so that it is not folded (Figure 5C).
20. Repeat step 19 twice.

***Note:*** The operator should complete steps 17 to 20 within 1 min; otherwise, the LIG will solidify before the experiment is completed.

17. Washing the 20 μL strippette tip 3 times with LIB. Then, gently aspirate the dissected pupal midgut (in LIB) under the microscope using a 20 μL pipette gun.
18. Place the live imaging dish under a stereo microscope and transfer the dissected pupal midgut (together with associated tissues in LIB) to the gap between the two coverslips using the pipette gun (Figures 5A and 5B).

21. Allow the LIG to solidify for 2 min at 25°C.
22. Add 20 μL of 37°C LIG to the space between the two 11 × 22 mm coverslips and quickly cover the midgut with a 22 × 22 mm coverslip, gently pressing the 22 × 22 mm coverslip with forceps to bring the coverslip as close to the midgut as possible (Figures 5D and 5E).
23. After 2 min, when the LIG has stabilized, seal the 22 × 22 mm coverslips with approximately 80 μL of halocarbon oil 27 using a 20 μL pipette gun to prevent water evaporation (Figure 5F).

### Live imaging of the pupal midgut under a confocal microscope

**Timing: ∼3 h, varies by experiment**

**Figure 6:**
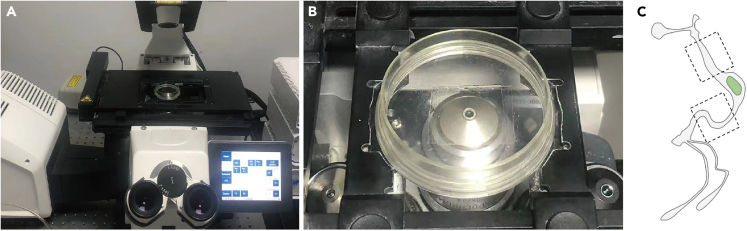
Live imaging of pupal midgut (A) Pupal midgut mounted on a coverslip and imaged by inverted confocal microscopy. (B) Zoom view of the imaged mounted dish. (C) Imaging areas are indicated by rectangles.
25. Image the mounted live imaging dish on a Zeiss LSM 800 confocal microscope equipped with a Plan-Apochromat 63x/1.40 Oil DIC M27 objective using the Immersol 518 F imaging medium (Figures 6A and 6B). Use definite focus to avoid focus drift, acquire time-lapse images using ZEN 2.1 with time-lapse module.
26. Take an image of the pupal midgut from the parts indicated by rectangles (Figure 6C).

***Note:*** The laser wavelengths of the green and red channels are 488 and 561, with a laser power of 2% and 0.2%, the detection wavelengths are 410-525 and 580–700, respectively, these channels are taken in frame, 8–12 μm z-stack images (2 μm intervals) of 512 × 512 pixels (0.198 μm × 0.198 μm) are acquired every 60 s with a pixel time of 1.03 μs.

24. The live imaging dish is placed without a lid, with the coverslip upside down so that the objective touches the coverslip (Figures 6A and 6B).

27. Adjust the total imaging time according to the requirements of the experiment and imaging.

***Note:*** Imaging the midgut can last for 3 h or longer. We choose 3 h because after 3 h of imaging we can hardly detect the pISC divisions This section images the ISC division in the *Drosophila* pupal midgut at 25°C. Experiments can also be performed at temperatures between 18°C and 29°C, if required.

28. When live imaging is complete, place the mounted dish in 50°C hot water for 5 min to melt the LIG, then rinse the dish with water, dry, and reuse.

**Figure 7:**
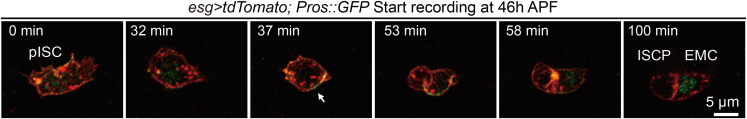
Asymmetric distribution of Pros::GFP during the pupal ISC division Single frames from time-lapse movies of an ISC undergoing asymmetric division. Red, tdTomato; green, Pros::GFP. Arrow indicates basal localization of Pros::GFP.

***Note:*** An example of live imaging of the pupal ISC division is shown in Figure 7. A non-inverted microscope can also be used for live imaging of the pupal midgut.

## Expected outcomes

The outcome of this protocol is a continuous video of the pupal ISCs.^1^ The frame rate and the duration of imaging can be adjusted to capture events for 3 h after dissection. Asymmetric division of Pros::GFP is observed in wild type progenitors. Gene knockdown or overexpression in the progenitor cells of the midgut may alter this process.

## Quantification and statistical analysis

**Timing: ∼1 h, varies by experiment**

This section outlines the quantification of the fluorescence in each cell manually using Fiji ImageJ for each frame throughout the timeline.

1. Carefully select the region of interest (ROI) based on the area of the cell (Figures 8A–8C, M1-M3).

**Figure 8:**
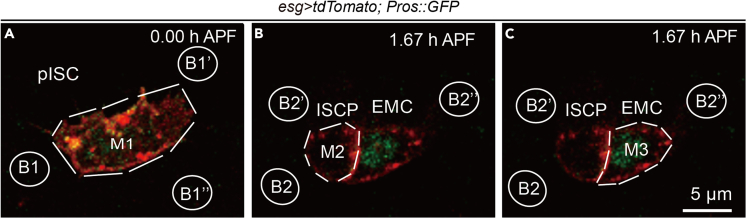
Select ROIs for quantification analysis (A) The ROI area selected in the progenitor cell (pISC). (B) The ROI area selected in one daughter cell (ISCp). (C) The ROI area selected in the other daughter cell (EMC). M1-M3 are ROIs, B1-B1″ and B2-B2″ are areas for background intensity determination.
2. Determine the area fluorescence intensity of ROIs in the green channel (of Pros::GFP) in projection images of recorded z-stacks, and exclude the background intensity.
3. Determine the average background intensity by selecting three individual regions adjacent to the ROIs (Figures 8A–8C, B1-B2).
4. Calculate the amount Pros in each cell by multiplying the area by the Pros::GFP intensity.

## Limitations

This is a live imaging protocol, so all image acquisition and analysis is dependent on genetically encoded fluorescently labeled proteins. This protocol is performed ex-vivo; therefore, the dissected intestinal tissue exhibits the ISC division for about 3 h due to the lack of in-vivo physical and physiological environment. Further optimization is needed for longer imaging.

## Troubleshooting

### Problem 1

Difficulty in dissecting the pupal midgut without disrupting it (Steps 10–16).

### Potential solution

Use a pair of blunt-ended forceps and a pair of fine-tipped forceps to facilitate dissection. The blunt-ended forceps are used to hold the pupa stable, while the fine-tipped forceps are used to dissect the midgut. The method of dissecting the pupal midgut is different for each developmental stage. The method given in this study is applicable to dissection of 46 h–52 h APF pupae (25°C). When dissecting pupae from earlier time points, such as 42 h APF, midguts are more likely to break when prying with forceps, using more pointed forceps will cause less compression of the gut, thus increasing the rate of successful dissection. In addition, when dissecting pupae earlier than 30 h APF, usually at the moment of forceps puncture of the abdomen, intra-pupal tissues gush out of the puncture as the intra-pupal organs are under positive pressure. At this point, LIB is aspirated using a pipette gun to rinse the mixed tissues, and the pupal midgut, which is at an early stage of development, can be distinguished by the green color of the meconium and transferred to the dish using the pipette gun.

For dissection of 80 h APF or later pupae, the dissection method is the same as for adult midgut dissection, and the midgut is transferred to the dish using forceps. At all times, try to avoid prying the midgut with forceps. To gain experience in dissection, it is recommended to practice dissecting the adult midgut first, then the late pupal midgut, and finally the early pupal midgut.

### Problem 2

The image is not clear (Step 27).

### Potential solution

The image will not be clear if the gut is far away from the 22 × 22 mm coverslip. When fixing the midgut with LIG, adjust the position of the midgut to be as close to the 22 × 22 mm coverslip as possible. One thing to keep in mind is not to use too much LIG to cover the midgut, which can cause the sample to be too far away from the 22 × 22 mm coverslip. Be careful to adjust the midgut to the correct position without damaging it.

### Problem 3

Difficulty in finding cell types at the correct development stage when imaging (Step 27).

### Potential solution

Selection of cell types at the correct developmental stage is based on the developmental time, cell morphology and Pros::GFP expression. For example, to study the first asymmetric division of stem cells, the pupa should be dissected at 46 h APF, when the stem cells are dispersed, cell spacing is relatively uniform, expression of Pros is low or not detectable, and the cell morphology changes from irregular to round. To study the symmetric division of enteroendocrine mother cells (EMCs), the pupa can be dissected at 52 h APF, when newly formed stem cells and EMCs are close together, Pros is absent from stem cells, and Pros accumulates in EMCs. Because live imaging only captures cell events in 3 h, it is important to image cells that are ready to divide. Finally, to ensure reproducible results, the culture temperature of the fly pupae should be as stable as possible.

### Problem 4

The pupal midgut is difficult to image for 3 h (Step 27).

### Potential solution

If the midgut is dying rapidly, as epithelial cells start to loosen, nuclei stop moving and intestinal muscles stop contracting, then increasing the speed and quality of dissection is likely to be needed to improve the integrity of the midgut and reduce the amount of time the midgut is not bathing in media. Shorter exposure times and lower laser settings may help reduce damage due to phototoxicity. If the pupal midgut drifts out of focus in the z-axis, manually bring the sample into focus. In addition, we have also developed an adult FlyVAB imaging strategy for long-term intravital imaging of the adult midgut.^7^

### Problem 5

Expression of the RNAi or protein of interest causes lethality before or during the pupal stage.

### Potential solution

The inclusion of *tub-Gal80*^*ts*^ is important if the gene of interest is lethal when expressed under *esg-Gal4* expression before the pupal stage. With *tub-Gal80*^*ts*^, it is possible to control gene expression by keeping the animals at 18°C for a longer period of time and minimizing the expression time of the gene at 29°C. One can also optimize gene expression levels by adjusting the culture temperature. To knock down genes before white pupa, collect white pupa at 29°C. The preparation of the live imaging dish of the pupal gut (from step 5 to 23, 20 min total), and the imaging process (Step 27) should be performed at 29°C.

### Problem 6

Lack of strong fluorescent-tagged proteins.

### Potential solution

If the protein of interest does not carry a fluorescent tag, or the fluorescent tag is weak, it may be necessary to construct a fluorescent tagged protein with higher fluorescence intensity. Alternatively, the introduction of two or more tags at the same time may result in increased fluoresce signal.

## Resource availability

### Lead contact

Further information and requests for resources and reagents should be directed to and will be fulfilled by the lead contact, Zheng Guo (guozheng@hust.edu.cn).

### Materials availability

All plasmids and fly lines generated in this study are available upon request to the lead contact.

### Data and code availability

• The data generated in this study are available upon reasonable request from the lead contact.
• This paper does not report original code.
• Any additional information required to reanalyze the data reported in this work paper is available from the lead contact upon request.
